# "Are we ready to transition from the Global Alliance for Vaccines and Immunization support?" Perceptions from 15 Kenyan counties

**DOI:** 10.11604/pamj.2024.49.29.45027

**Published:** 2024-10-04

**Authors:** Alex Olateju Adjagba, James Odhiambo Oguta, Elvis Omondi Achach Wambiya, Caleb Nyakundi, Sharonmercy Okemwa, Catherine Akoth

**Affiliations:** 1 University of Western Cape, Cape Town, South Africa,; 2 Health Section, UNICEF, Eastern and Southern Africa Regional Office, Nairobi, Kenya,; 3 Division of Population Health, Sheffield Centre for Health and Related Research, School of Medicine and Population Health, University of Sheffield, Sheffield, United Kingdom,; 4 Moi University, School of Nursing, Eldoret, Kenya

**Keywords:** GAVI Alliance, immunization, funding, Kenya, transition, county, subnational

## Abstract

**Introduction:**

Gavi, the Vaccine Alliance, defined a transition roadmap for countries receiving funding support based on their income status projections. According to the latest projections, Kenya will complete their transition from vaccine funding in 2029. While eligible countries are kept informed and supported for a smooth transition process, the extent to which countries understand the significant implications of a complete end of GAVI support on immunization service delivery varies. Furthermore, whereas studies have been conducted to assess national preparedness for transition, there is a paucity of data on the understanding of subnational authorities of this process. In this study, we explored the perspectives of county-level stakeholders on Kenya's preparedness for GAVI transition.

**Methods:**

using purposive sampling, 77 senior county officials from 15 counties were selected for in-depth interviews. Data were collected using a semi-structured interview guide, transcribed, and thematically analysed. Ethical approval for the study was granted by Moi University Institutional Ethics and Research Committee.

**Results:**

findings reveal a consensus among respondents that both national and county governments are not fully prepared for the end of the Gavi Alliance. Concerns were highlighted around a lack of knowledge about vaccine costs, post-transition funding sources, and potential disruptions in immunization services. Respondents advocated for a phased transition, continued donor support, clear funding allocation, and legislative measures to ensure financial sustainability. Moreover, advocacy and awareness efforts, capacity building, and a robust legal framework were emphasized as essential for a smooth transition.

**Conclusion:**

after the end of the financial support provided by Gavi Alliance, Kenya's immunization bill is expected to be significant. This study underscores the importance of effectively engaging the subnational (county) level authorities. Successful transition from Gavi's support requires a strategy that promotes awareness and improves communication regarding the expected impact of the impending transition from Gavi on sustainable immunization financing in Kenya.

## Introduction

Gavi, the Vaccine Alliance, was established in 2000 to support countries financially and programmatically in expanding immunization programs and to ensure that countries can sustain themselves once Gavi support ends [1]. There is strong evidence that the support from Gavi has helped improve immunization coverage and reduce child mortality related to vaccine-preventable diseases across the world. For every dollar per capita invested by Gavi, there is an associated decrease in mortality rate by 1.8 per 1000 live births annually [2]. For instance, following the introduction of the pneumococcal conjugate vaccine, Kenya experienced a significant decline in invasive pneumococcal disease from 81.6 per 100,000 to 15.3 cases per 100,000 [3]. Currently, the immunization coverage for the third dose of Diphtheria-Pertussis-Tetanus vaccine (DPT3) is 89.2% [4], with donor assistance contributing to improved coverage of measles, pneumococcal, pentavalent, and rotavirus vaccines. However, there is a notable regional and socioeconomic disparity in immunization coverage. As an example, for DPT3, there is a 72.7% difference between the highest coverage in Nakuru and the lowest coverage in Garissa [4].

Gavi aims to focus its support on the world's poorest countries and therefore, bases eligibility on national income. In 2024, countries become eligible for Gavi support if their most recent gross national income (GNI) per capita was less than or equal to US$ 1,810 (according to the latest World Bank data published in July each year). Since 1^st^ January 2011, the eligibility threshold has been adjusted for inflation on an annual basis. Countries transition to the accelerated phase once their three-year average GNI per capita surpasses the threshold, after which Gavi reduces its funding support [5]. The accelerated phase lasts eight years, and the countries become fully self-financing [6]. Although a country may have achieved the threshold set for self-financing, the country's underlying situation may not necessarily change [5]. This transition approach does not consider the context and performance of health systems; therefore, preparedness for financing and institutional capacity is not captured.

Based on the Gavi financing model, Kenya entered the accelerated phase of support in 2022 and is still eligible until 2029. Vaccination costs are projected to increase from $10 million in 2023 to $38 million by 2029 [7]. The treasury has previously failed to timely disburse co-financing payments to Gavi and plans toward sustainable domestic financing are challenged by uncertainty in vaccine prices [8,9]. To provide financial flexibility during the transition period, the country relies on the Vaccine Independence Initiative (VII), a collaboration with UNICEF, to ensure an uninterrupted supply of vaccines and meet co-financing procurement requirements. However, VII may not adequately meet the expanding need for immunization [8]. Despite support over the years, immunization programs continue to face vaccine stockouts due to supply chain disruptions, poor logistics systems, and poor forecasting [10,11]. Moreover, there is reduced funding for immunization programs both at national and county levels, along with significant budget cuts for the health sector.

To manage the transition from Gavi support effectively, Kenya must deal with programmatic risks, including the support for vaccines, support for immunization systems, access to preferential vaccines and procurement channels, and technical and managerial support [12]. Countries that have graduated from Gavi assistance have improved or successfully maintained key outcomes compared with their expected performance [5]. Countries with a stronger institutional environment and more effective government administration were more successful in transitioning. However, countries transitioning under the current transition policy have seen a drop in immunization coverages [13]. Angola still faces gaps in programmatic capacities since graduation in 2021 with decreasing immunization coverage rates and is therefore still benefiting from the Gavi targeted support to mitigate the challenges [14,15].

Poorly executed transitions, especially in large-scale health programs, risk reversing health achievements such as immunization coverage and negatively affecting services and outcomes [16]. With Kenya preparing to transition from Gavi support, there is a need to evaluate the country's readiness to sustain immunization programs independently. This qualitative study addresses the gaps in understanding Kenya's preparedness by focusing on programmatic risks, the capacity of institutions, and the challenges associated with the transition.

## Methods

**Study setting:** this study was conducted in Kenya, which is a lower middle-income country situated in Eastern Africa [17]. Kenya has a devolved system of governance consisting of the national government and 47 semi-autonomous county governments. Healthcare is a function of both levels, with the national government playing a policy and regulatory role, while the county governments are in charge of health service delivery and implementation at the community level. The national government oversees vaccine procurement and distribution to regional depots. Immunization program implementation and service provision at the county level is coordinated by the Expanded Program on Immunization (EPI) logisticians in liaison with the National Vaccines and Immunization Program (NVIP).

**Sampling:** for this study, counties were grouped into eight regions as per the former provinces of Kenya. Counties were then selected based on immunization coverage for the period 2014-2017. In consultation with the NVIP management, counties were purposely selected. The following 15 counties were selected based on immunization coverage: Bomet, Bungoma, Uasin Gishu, Kakamega, Kiambu, Kisii, Kisumu, Makueni, Murang'a, Mombasa, Nairobi, Nakuru, Taita Taveta, Tharaka Nithi, and West Pokot. Participants were also purposely recruited from the selected counties based on their roles in planning, management, and implementation of health programs, including immunization at the county level. Snowballing approach was further used to identify relevant officials within the county departments. A total of 77 participants were recruited and interviewed as summarized in Table 1.

**Table 1:** list of participants

Position Held	Number Interviewed
Other County Health Management Team Members	20
County Executive Committee Member (Health)	3
Director of Nursing Services	2
County Director of Health/Public Health/Medical Services	13
Chief Nursing Officers	5
Chief Officer of Health/Public Health/Medical Services	7
Expanded Program on Immunization Logisticians	15
CDOH Accountant/Economist/Finance	12
**TOTAL**	**77**

**Data collection and analysis:** data was collected through in-depth interviews with 77 key county health officials from the 15 selected counties using a semi-structured interview guide. Verbatim transcription was performed on the audio-recorded interviews. An inductive thematic analysis approach was conducted, guided by the Braun and Clarke framework [18]. To ensure accuracy and completeness, the transcripts were read and reread multiple times. The transcripts were coded to identify emerging parent and child codes using Nvivo 14 Software. The generated codes were compared to maintain consistency and agreement among the researchers. A codebook was created by integrating and collating these codes, after which overarching themes and subthemes were identified and reviewed to confirm their suitability for interpretation.

**Ethics statement:** ethical approval to conduct this study was granted by Moi University Institutional Research and Ethics Committee (Approval Number: 003605). Informed consent was sought from all participants before conducting the interviews, and the transcripts were anonymised at reporting.

## Results

**GAVI transition readiness:** there was a consensus among respondents that both the national and county governments were not fully prepared for the transition away from GAVI funding, which will potentially lead to financial constraints.

"*For the GAVI transition, I think our country and this county are not ready. We are still struggling even with buying these drugs and these other commodities. For example, if today county governments are to buy only the syringes and GAVI still supports the vaccines, it is still a struggle. So, I think we need to rethink how we will support the immunization program*." KI 65.

"*Gavi should not transition in supporting countries like Kenya, however much we have achieved middle-income status. They still have to support these countries in attaining the targets that we had set previously*." KI 061.

"*It is unfortunate, first of all, that Gavi is transitioning. In my opinion it should not commence the process. However, if it does happen, I think we have to change the way we fund our counties especially for essential expenditures like purchase of medicines and medical supplies*." KI 052.

**Anticipated challenges associated with the GAVI transition:** the respondents indicated that Kenya will likely face challenges both at the county and national levels with the GAVI transition. Challenges of financial constraints emerged significantly, with concerns of the availability of funds once GAVI support ceases.

"*At the moment, it means that the county governments and even the national government rely on donors to fund the program. You see, it seems that we are not yet able to fully support these programs. So, maybe, I do not know where the governments will get funds alone, because it will mean now that we will need to solicit for another donor or request the same donors to continue funding the program. Because as it stands now, most of our programs are funded by donors, especially on health matters*." KI 002.

"*We have the vaccines but even now and then, we are running short of the syringes because the SoloShots are not found in every chemist*." KI 009.

"*Like I have told you, sometimes there are no funds. So, if there are no funds, there are not enough vaccines and now if even syringes and needles are an issue, how shall we manage?…*" KI 021.

Most respondents highlighted that the health sector in Kenya is already overburdened and relies majorly on donor funding. The withdrawal of vaccine funding would limit access to immunization services, leading to the possibility of people having to pay for vaccines, given the uncertainty about National Health Insurance Fund (NHIF) support for immunization programs.

"*So, what I know is that without support from those multinational relief the government may not be able to put enough money to bring the vaccines here*" KI 025.

"*Vaccines are expensive, it means now that people have to dip, they will have to either maybe the county allocates enough money to the health department or maybe now the NHIF could cover immunization…*" KI 049.

Respondents also expressed that the GAVI transition would leave gaps in terms of logistics, which included delays in procuring, handling, and management of vaccination. Respondents further expressed concerns about the complexity of the vaccine logistics.

"*I anticipate the problem of getting those vaccines…making sure that they are paid for…given that we have not paid for them ourselves in the past and I am imagining it is a long process…so that is a challenge*." KI 021.

"*I think I am a bit worried because you know, the Kenyan system is always up and down and knowing that immunization is not charged, we anticipate just like we have challenges when it comes to other commodities, we expect that stock outs will be more when GAVI withdraws. We expect more delays in procurement because these are common things, we are seeing so it is a source of worry to most of us yeah*." KI 054.

"*You know we have issues of power, power outages and the rest. So, we really need to have serious guys trained on how to handle the vaccines the way they have been handling in the depots. And then of course, the storage equipment, we need to be sure of that first*." KI 001.

"*The transition is going to be about technical capacity building, infrastructure and also expanding the accessibility of the service up to the lower level to the community level so that means probably increasing the number of facilities or opening up new outlets as opposed to that is available so that we reach as many children as possible*." KI 036.

Other concerns with the GAVI transition included lack of knowledge about vaccine costs and the procurement processes.

"*Like now we don't know the cost of vaccines, we don't. So, we may need to be given the information, so we know that if I need to procure a vaccine, I know the total amount and where we procure from. So, all we need is time and a good time*." KI 031.

"*I don't know whether it is the problem with Tharaka-nithi or the other counties one as a county we don't know how much we get from Gavi with all the prices of these vaccine we don't know so today even if we are told to budget we may not be able to say this is the requirement for the procurement of vaccine, so that is a challenge*." KI 053.

Most respondents highlighted the potential disruption in immunization services that would occur because of the transition. Some of the short and long-term impacts highlighted included the re-emergence or increased burden of vaccine-preventable disease.

"*I think they need to be careful because if they just do it like that, I'm very sure there are places where it won't go for long before those diseases of old age start coming up and then it will become another problem again, yeah. Especially when we talk about these marginalized areas…*" KI 051.

"*Imagine if we fail to immunize our children, we will be having a generation that will not be healthy at all. There will be all those diseases that we have overcome, and they will be coming back*." KI 021.

**Recommendations for sustainable transition:** with the realization that the transition is inevitable, the respondents proposed measures that will ensure successful transition processes and sustainability post-GAVI exit.

***Advocacy and awareness:*** advocacy efforts were cited to ensure that both national and county governments understand the importance of immunization programs and are prepared for the funding transition. This is crucial for the success of immunization programs post-transition. In addition, awareness campaigns are needed to inform stakeholders about the costs of vaccines, the importance of immunization, and the potential consequences of funding changes. Respondents also emphasized the involvement of county assemblies in the decision-making process related to immunization funding and planning.

"*A recommendation is that GAVI does a lot of high-level advocacy with the county governments so that everybody sees the sense of good planning on immunization*." KI 054.

"*There is advocacy, there is a lot of political goodwill, there is a lot of awareness that needs to be done for a smooth transition*." KI 058.

"*I believe that before Gavi pulls out, or before any, it's good that we sensitize everybody, the whole of the county government needs to be sensitized to know the implication of a partner pulling out*." (Taita Taveta EPI).

"*For example, if probably Gavi decides that Kenya should purchase antigens for immunization, we really need to be taught on how to go about that yes. Otherwise, immunization services might get to a standstill because probably the politicians might not see the importance of that. We really need to be sensitized from the top to the bottom*." KI 019.

"*There is also a need to sensitize all the stakeholders so that they are aware, including the county assembly, because they are the approvers of the budget*." KI 003.

***Gradual transition:*** to ensure a successful transition, respondents suggested that the transition be phased rather than an abrupt cessation of funding. This demonstrates a low level of awareness about the current processes which are already gradual. Respondents also raised concerns about the government's capacity to fully run the immunization program independently, indicating a potential gap in financial resources that may need to be addressed through continued partnership or innovative financing mechanisms.

"*If it has to happen, if it must happen let it be gradual. Gradual in a sense that it is agreed so that people understand so that people prepare and put a budget for vaccines*." KI 054.

"*I think what should happen first, the support should not end abruptly, we phase it out. So, they start from some components as the county prepares to take over…*" KI 001.

"*I hope the transition process should be negotiated in a proper way, and everybody is aware and therefore it is not going to be an abrupt transition. So, once it is properly planned, then the government will just have to pick the program and provide the allocation like any other activity*." KI 024.

***Planning and budget allocation for immunization:*** respondents stressed the needs for adequate time to plan for the transition and budget allocation to counties for vaccine procurement to ensure that counties can plan and budget effectively, minimizing the risk of stockouts or disruptions in immunization services.

"*The county should be given enough time to plan, and it should not be done like tomorrow… We need time*." KI 031.

"*We also need to train the technical officers in the counties on doing multi-year plan for commodity immunization commodity multi-year plan so that you can see ten years what the budget should be like using the projections of the population so that it becomes easy with that multi-year plan it will be easier to plan*." KI 054.

"*In my own opinion, all the stakeholders need to sit down and put down strategies and see how to come up with a budget that will cover immunization since the partner, GAVI will be exciting*." KI 004.

"*We also need to prepare the counties that very soon donors will be pulling away and we may need full financing. So, they better plan to accommodate immunization as an important preventive component in their budgets*." KI 005.

Regarding county-level funding and accountability, respondents proposed that resources allocated to immunization be ring-fenced with the aim of enhancing accountability and transparency in financial management. Furthermore, there was an emphasis on the need to institute legislative measures to mandate counties to allocate resources specifically for immunization programs.

"*Like I said, the flow of funds should be very clear, and the proper system should be put in place so that we also sustain vaccine stock because my fear is that if the counties are left to handle vaccines, I think we will have some erratic stock out and the rest*." KI 027.

"*Vaccines should be accommodated in the national annual budget. I think they should factor it in the national budget because it needs a very huge sum of money. So, I think it should be factored in at the national level and this should be handled by the national government*." KI 007.

"*I think there should be re-fencing of money for specific programs that have immunization included so that this money, when it comes to the county, cannot be used for any other thing but for the purposes of running the immunization*." KI 048.

"*I think they need to have legislation to force counties to prioritize the areas that they need to allocate resources…*" KI 061.

"*We need to have a legal framework to protect immunization*." KI 053.

***National Government responsibility and oversight:*** respondents expressed varying views on the role of government, particularly the county versus national government, in immunization programs after the GAVI transition. Some recommended that vaccine procurement remain the national government's responsibility for quality control, coordination, and distribution of vaccines. Some expressed concerns about the capacity of the county government to manage vaccine procurement effectively and enhance adherence to quality standards.

"*I would recommend that the function of procurement be left with the national government, for the quality checks and coordination, so that we are not offering different services as counties. I wish that it remains a function of the national government because all the technocrats are there. Remember, a lot of decisions here are affected politically*." KI 005.

"*I would say let the national government handle that issue. National government and not county government because I think we are having issues with county government. They don't understand some of these projects…*" KI 050.

## Discussion

Like several other lower middle-income countries, Kenya will soon be expected to take over the full cost of immunization programs. In this study, we qualitatively assessed the readiness of the government to transition by interviewing key informants. Respondents expressed doubts about the government's preparedness for GAVI transition, highlighting gaps in knowledge about vaccine costs and uncertainties regarding future funding sources. These concerns suggest a lack of comprehensive planning and a potential disruption in immunization services post-Gavi transition. The provision of oversight and guidance through policy, regulation, system design, and accountability is very crucial during or following the donor exit [19]. Previous studies have highlighted that lack of government readiness could affect program continuity, leading to gaps in health financing and technical capacity and disruptions in health service delivery in the post-transition period [20,21].

Potential challenges associated with the Gavi transition were highly emphasized. Financial constraints were a primary concern, and respondents expressed concern about the availability of sufficient budget allocations. Kenya's health sector is already overburdened and reliant on donor funding and may struggle to maintain essential services like immunization without external support [22]. Additionally, political factors influence the allocation of domestic resources, which can affect which regions receive sufficient funding and support [23]. Countries graduating from Gavi funding require sustained financing of health services backed by strong political commitment and technical capacity to effectively plan and manage immunization programs for financial self-sufficiency [24]. A lack of political will coupled with inadequate funding has been shown to contribute to catastrophic effects in Romania, Serbia, and Albania, resulting in the collapse of programs such as the HIV prevention post-donor exit [25]. Even with political will, governments may find it difficult to close the funding gap due to limited domestic resource mobilization capacity [26].

Logistical challenges were also underscored, including procurement delays and vaccine management issues. The complex logistics of vaccine distribution and potential issues like power outages affecting storage add another layer of possible difficulty post-transition. For example, countries transitioning from Gavi support faced challenges in paying higher prices for direct vaccine procurement compared to Gavi-eligible countries, with costs nearly twice as high in some cases [12,24]. Some countries also experienced supply chain challenges due to losses in technical capacity for procurement post-donor exit. For instance, Bangladesh was unprepared to take on the procurement of antiretroviral medications after the Global Fund's departure. Donors offering technical support or capacity building throughout the transition process ensured a smoother transfer of procurement responsibilities [20]. Where safeguards like technical capacity and government commitment were present, countries sustained procurement practices effectively post-transition. For example, Albania self-financed its domestic vaccination programming without donor support, avoiding vaccine stockouts or shortages [21]. Other countries integrated system-wide changes, such as new policies or regulations, into the transition process, ensuring sustainable funding for health programs [25,27].

Different strategies that can be adopted to maintain the financial sustainability of health programs during and after the withdrawal of donor support were proposed. Respondents called for high-level advocacy within county governments to highlight the significance of building technical capacity for effective planning and management of immunization programs. Moreover, advocacy efforts can ensure all stakeholders, including county governments, understand the importance of sustained immunization funding and are adequately prepared for the transition. Several respondents underscored the necessity for enhanced training and education on vaccine procurement and purchasing processes, thus emphasizing a critical gap in current capabilities. This is essential to prevent potential post-transition failure, as seen in Botswana, where, despite financial preparation from the World Food Program, technical capacity gaps after the transition led to reduced program efficiency [28]. Similarly, the transition of the U.S. The President's Emergency Plan for AIDS Relief (PEPFAR) in South Africa highlighted the negative impacts of poor planning and short transition timeframes, resulting in disruptions to treatment regimens [20].

Engaging community health stakeholders and participation of county assemblies in decision-making related to immunization funding and planning were emphasized, ensuring all relevant parties were informed and engaged in the transition process [29]. According to Resch *et al*. [30], for a transition to be successful, it is essential to conduct a readiness pre-assessment, establish a jointly agreed-upon transition plan between donors and recipient countries, implement a framework to monitor the transition process and create mechanisms to ensure accountability [30].

Adequate health financing is a product of proper mobilization of funds and efficient allocation of resources to ensure that the health systems have sufficient funding for equitable access to high-quality health services [31]. Donor transitions impact health financing within countries, influencing not only health program budgets but also financial management capacity, service delivery, and human resources [32-34]. As stressed by respondents, multi-year budgeting will provide counties with sufficient time to plan and allocate resources, thereby minimizing the risk of stockouts or disruptions in immunization services. This highlights the need for a robust financial framework that includes immunization as a critical component of the health budget. However, there were concerns about the government's capacity to fully finance the immunization program independently, indicating potential gaps that may need to be addressed through continued partnerships or innovative financing mechanisms. Previous studies have highlighted gaps in planning and budgeting for immunization at the subnational level in Kenya [35] and the need for equitable distribution and effective coordination of relevant technical support from partners to improve immunization financing [36].

Respondents proposed ring-fencing funds for immunization programs at the county level to enhance accountability and prevent the reallocation of resources to other departments. Earmarking of funds ensures financial sustainability for specific initiatives after donors' exit [37,29]. For instance, the Albanian government ensured financial sustainability for its vaccination programs post-Gavi by establishing a separate budget line for vaccines [21]. Similarly, in North Macedonia, the Ministry of Health registered Non-governmental Organisations (NGOs) for government funding eligibility for HIV prevention, creating a social contracting mechanism post-donor exit [38]. In addition to earmarking, other governments have devised innovative methods to mobilize domestic resources to fill the funding gaps left by donor withdrawals. For example, Kenya introduced a levy on airline tickets and passed a bill in 2012 to allocate 1% of tax revenue towards bridging the domestic financing gap for HIV/AIDS programs [37,39]. Such initiatives may apply to the Gavi transition. However, insufficient preparation and commitment can lead to significant financing gaps, posing sustainability challenges [40,41].

**Strengths and limitations:** these study results shed new light on the perspectives of the Gavi transition from a diverse group of respondents at both national and county levels. However, the findings are limited to the generalizability to other contexts, potential biases from self-reported data, and a narrow focus on the Gavi transition.

## Conclusion

Kenya's readiness for the Gavi transition poses challenges that require comprehensive planning, adequate funding, and robust capacity building beyond the usual national-level structures. A phased transition, starting from subnational levels supported by clear funding allocation, legislative measures, and strong advocacy, is crucial for sustaining immunization programs post-Gavi. Addressing financial, human resource, and logistical challenges in support of county-level planning and budgeting through continued partnerships and innovative financing mechanisms will be essential for a successful transition and the continued protection of public health.

### What is known about this topic

- Gavi, the Vaccine Alliance, financially supports low-income countries, including Kenya, with vaccines and immunization programs;
- Kenya is transitioning to self-financing for vaccinations with the imminent exit of Gavi by 2030.

### What this study adds

- This study reports county health officials' perceptions of county preparedness for Gavi transition and their recommendations for a sustainable transition;
- These findings are in favour of shifting partner and Gavi support beyond the usual national-level structures.
